# Edaravone Improved Behavioral Abnormalities, Alleviated Oxidative Stress Inflammation, and Metabolic Homeostasis Pathways in Depression

**DOI:** 10.1155/2023/6623141

**Published:** 2023-07-18

**Authors:** Sina Montazeri, Soroush Bijani, Mahdieh Anoush, Ali Sharafi, Ali Kalantari-Hesari, Mir-Jamal Hosseini

**Affiliations:** ^1^Zanjan Applied Pharmacology Research Center, Zanjan University of Medical Sciences, Zanjan, Iran; ^2^Zanjan Pharmaceutical Biotechnology Research Center, Zanjan University of Medical Sciences, Zanjan, Iran; ^3^Department of Pathobiology, Faculty of Veterinary Science, Bu-Ali Sina University, Hamedan, Iran

## Abstract

Depression is one of the main factors affecting our daily performance. Among many putative compounds with effect on behavioral and pathophysiological alterations in depression, edaravone (EDV) demonstrates antioxidant and free radical scavenging properties. To investigate possible antidepressive and anxiolytic-like effects of EDV, Wistar rats were randomly divided into six groups: (1) control; (2) EDV (6 mg/kg); (3) post weaning social isolation (PWSI); (4) PWSI+EDV (1.5 mg/kg); (5) PWSI+EDV (3 mg/kg); and (6) PWSI+EDV (6 mg/kg). After the series of behavioral tests, animals were sacrificed, and their hippocampi were dissected for further biochemical and gene expression assays. Our results showed that treatment with 3 and 6 mg/kg EDV after social isolation would improve anxiety, depressive and anhedonic-like behavior in OFT, EPM, FST, and splash tests. In addition, treatment at the aforementioned doses achieved to recover total cellular antioxidant and GSH level. These effects were accompanied with the suppressive effect of EDV on MDA and PCO levels. EDV treatment also modulated the expression of *AMPK*, *Tlr-4*, *BDNF*, *nNOS*, and *iNOS* genes. The treatment with 3 and 6 mg/kg EDV would lead to the recovery of behavioral impairments, cellular free radical surge that could be in correlation with the effect of this substance on immune system response, improved energy production system, and more efficacy in the recovery of neural tissue. In conclusion, EDV ameliorates depressive-like disorder by modulating neuroinflammation, energy production, and neural tissue recovery.

## 1. Introduction

Depression is one of the leading causes of a lack of expected social performance among individuals, as 80% of adults have difficulty in their vocational and social interactions [1]. Depression is considered a lifelong disease instead of an acute illness which contributes to functional impairment in individuals and lower level of productivity in the community [2]. These patients report more problems in their social interaction and require more effective emotional stimuli in order to engage in social interaction [3]. Risk factors such as gender discrimination, disability, loss of kin members, and poverty could cause chronic loneliness. These problems cause the quality of life for over 15–30% of the general population [4, 5].

Social isolation is one of the main reasons for depression. Today, having satisfaction in social interaction would be one of the main aspects of welfare [6]. Social seclusion could lead to three main mental disorders, namely, depression, social anxiety, and paranoia, which may cause obesity, neuropathic pain, exacerbation of neurodegenerative disorders, and disorder in the hypothalamic-pituitary-adrenal axis (HPA-axis) [7]. Social isolation stress (SI) in rodents is a model of chronic stress early in life and is capable of producing a variety of behavioral changes that mimic depressive symptoms in patients [8]. SI could cause alterations in the pathological structure of brain inducting, significant microglia loss [9], and downregulation of brain-derived neurotrophic factor (BDNF) which resulted in a significant decrease in pyramidal CA1 cell layers and neuronal density in dentate granule [10].

Nitric oxide is one of the main factors that rise during chronic stress condition. The accumulation of NO can lead to an increase in level of reactive nitrogen species, causing impairment in mitochondrial performance and cellular energy production. The consequent event can lead to the malfunction of the HPA-axis, overproduction of proinflammatory cytokines, and microglial activation [11]. The expression of inducible nitric oxide synthase increases in traumatic conditions and expresses mainly in microglial cells [12]. The most affected parts of the brain by neural nitric oxide synthase (nNOS) expression are in GABAergic interneurons and glutamatergic pyramidal cells. The lower expression level of nNOS can be related to the activation of cellular death signaling pathways and increase immunoreactivity response [13].

Edaravone (EDV) is a neuroprotective and scavenger of free radicals, which first approved for amelioration of amyotrophic lateral sclerosis in 2017 [14]. The main underling mechanism consists of antiapoptotic properties of this substance would have a boosting effect on BCL-2 expression by demonstrating mitochondrial functional recovery, which is achieved by ATP production modulation and inhibiting mitochondrial swelling during accumulation of reactive oxygen species in neural cell [15, 16]. EDV can modulate HPA-axis function and gene pathways related to depression [17, 18].

In this study, our main goal is to investigate the possible neuroprotective effects of EDV against the behavioral and biochemical alterations caused by SI in rats. The factors regarding self-care, anxiety, and depressive-like behavior were measured. In addition, the effect of EDV on the endogenous antioxidant level and the expression of various inflammation marker genes were also investigated. We expect EDV can improve antioxidant levels and cellular homeostasis.

## 2. Materials and Methods

### 2.1. Animals and Housing Conditions

A total number of 60 male Wistar rats in the postnatal stage of development (PND: 21-23) were housed under standard laboratory conditions with free access to food and water for five weeks in social condition (control) and postweaning social isolation (PWSI). Socially conditioned rats were kept in groups, and PWSI rats were housed individually in a separate room (four rats per cage). The ages of PWSI rats were cleaned only one time per weeks by the same experimenter to avoid minimal handling and social contact. Wood shavings were used as bedding for animals. Animals were housed under 12 hour's cycles of artificial day and night, under temperature of 21-23°C and 50-55% humidity without any limit on their access to food and water. Following the NIH guidelines and ethics committee of the Zanjan University of Medical Sciences (IR.ZUMS.AEC. 1401.014), every intervention and procedure were performed between 10 : 00 AM and 02 : 00 PM.

### 2.2. Materials

The analytical grades of each solvent were purchased from the Sigma-Aldrich Company (Taufkirchen, Germany). EDV and glutathione were purchased from Merck & Co. The anesthetic agents (ketamine–xylazine) were purchased from Alfasan Co. (Woerden, Netherlands).

### 2.3. Animals and Treatments

**Figure 1:**
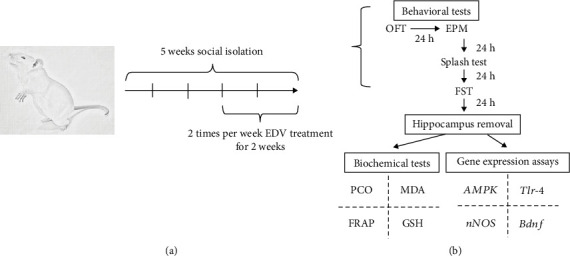
The timeline of the depression induction procedure, treatment, behavioral, and molecular assessment (a). The construction of the open field test, elevated plus maze, force swimming test, and splash test, respectively, (b).

Animals were randomly divided into 6 groups; each group housed 10 rats. The first group (control) was under normal social condition and treated only with normal saline once a day for two weeks. The second group was also under a normal social condition but was subjected with 6 mg/kg of EDV via an intraperitoneal injections (IP). Due to the hydrophobic, long effect and high-neural tissue delivery of EDV, the injections were separated by 3-4 day intervals (two times per week for two weeks) and performed during 10 AM to 2 PM. The third group (PWSI) was under isolation condition for two weeks then subjected to normal saline two times per week via an IP route. The fourth, fifth, and sixth groups (PWSI+EDV 1.5, 3 and 6 mg/kg) were under isolation condition for two weeks, then injected with 1.5, 3, and 6 mg/kg of EDV via an IP route two times per week. Twenty-four hours after the behavioral tests (FST), animals were sacrificed, and the hippocampus of the rodents was dissected [19]. The obtained tissues were frozen at -80 for further biochemical and gene expression assays (Figure 1 depicts the experiment design).

### 2.4. Behavioral Tests

#### 2.4.1. Open Field Test (OFT)

To measure the effect of SI and the possible effect of EDV on locomotor activity, OFT was utilized. The OFT maze consists of a white box made out of opaque plexiglas (50 × 50 × 40 cm). Its surface was divided into 16 equal squares. Animals were carefully placed in central zones, and the horizontal activity (as the number of crossed squares), vertical activity (the number of time rats stand on their feet, hands on the wall), and central zone locomotion (CLZ) were assessed within the analyze span of 5 minutes [20].

#### 2.4.2. Forced Swimming Test (FST)

One of the main behavioral tests used to measure despair behavior in rodents is the FST. This test consists of a cylinder (10 × 25 cm, diameter × height) with a level of 19 cm of water at 23 ± 1°C. Animals were placed on the surface of water, and for a span of 5 minutes, the time during which rats made no attempt to rescue themselves was measured [21].

#### 2.4.3. Splash Test

This test is designed to measure motivational and self-care behavior in rodents by assessing their willingness to groom and clean 10% sucrose solution from their bodies which was sprayed on the nape of the rodent. The self-grooming activity was analyzed in the 5-minute span of recording separately for each rat [22].

#### 2.4.4. Elevated Plus Maze (EPM)

EPM test was utilized to evaluation of anxiolytic behavior in rodents. This maze consists of four arms, two closed ones, and two open ones which standing faced to each other. The lighting condition of environment were bright so the rats could choose to stay in exposed or covered arms. Rats were placed in the open and exposed ones. This behavior was analyzed in each rodent for the span of 5 minutes. The percentage of entry to open arms is defined, as the time rats spent in open arms divided by the total analyzed time [23].

### 2.5. Biochemical Assays

#### 2.5.1. Lipid Peroxidation and Protein Carbonylation

A high level of polyunsaturated fatty acids and numerous proteins is located in neural cells. This makes these cells more susceptible to the escalating pattern of free radicals, and peroxidation and carbonylation level can be used as a marker for measuring tissue's free radical alterations. The lipid peroxidation (LPO) assay is based on the reaction of malonaldehyde (MDA) with thiobarbituric acid (TBARs) with highest absorption at 532 nm. The protein carbonylation (PCO) assay is based on the reaction of carbonylated proteins with 2, 4 dinitrophenyl hydrazine (DNPH) at 365 nm [24].

#### 2.5.2. Ferric Reducing Antioxidant Power (FRAP)

FRAP is the direct representor of the total cellular antioxidant, which accumulation of free radicals significantly drops the FRAP value. This test is based on the reaction of cellular antioxidants with Fe^3+^ (2,4,6-Tri(2-pyridyl)-s-triazine) solution at 593 nm [25].

#### 2.5.3. Redacted Glutathione Assay (GSH)

GSH is one of the main endogenous antioxidants that would encounter with oxidative stress escalation. This assay is based on the reaction of this antioxidant in reduced form with 5,5′-dithiobis(2-nitrobenzoic acid) at 412 nm [26].

#### 2.5.4. Gene Expression Assessments

To extract RNA from hippocampus samples, TRIzol reagent was used. Changes in expression level were measured by the real-time PCR method. 1 *μ*g of the extracted RNA was utilized for the reverse transcription process using a Prime Script RT reagent kit. The list of forward and reverse primers used in this experiment is mentioned in Table 1. SYBR Premix Ex Taq technology (Takara Bio) was applied for the thermal cycling process. Terminal steps performed in this experiment are as follows: 30 seconds at 95°C for initial activation and 45 cycles of denaturation and simultaneous extension/annealing for, respectively, 5 s at 95°C and 20 s at 60°C. To normalize the output and fold alterations in expressions, *β* actin was used, and the expression related to *β* actin was calculated using the 2^-*ΔΔ*ct^ formula.

#### 2.5.5. Histopathological Assessment

After performing cervical dislocation on rodents, the total extracted brain was fixed in 4% buffered formalin and stored for further exams. The dehydration process was done using sequential dehydration using 50, 70, 80, 90%, and absolute alcohol. After the clearing and paraffin fixation of the tissue, 5-7 *μ*m slices were prepared and installed on slides. Hematoxylin and eosin staining (H&E) was performed, and the pathological alterations were studied under an optical microscope [27].

#### 2.5.6. Statistical Analysis

Results were expressed as mean ± SD, and R studio programming software was used for statistical analyses. Comparison between the groups was performed using a one-way analysis of variance (ANOVA) followed by Tukey's post hoc, and a *P* value lesser than 0.05 was considered statistically significant. Plots were generated using the Matplotlib library in Python.

## 3. Results

### 3.1. Edaravone Did Not Alter Horizontal Activity While Improving Vertical Activity and Central Zone Locomotion in OFT

**Figure 2:**
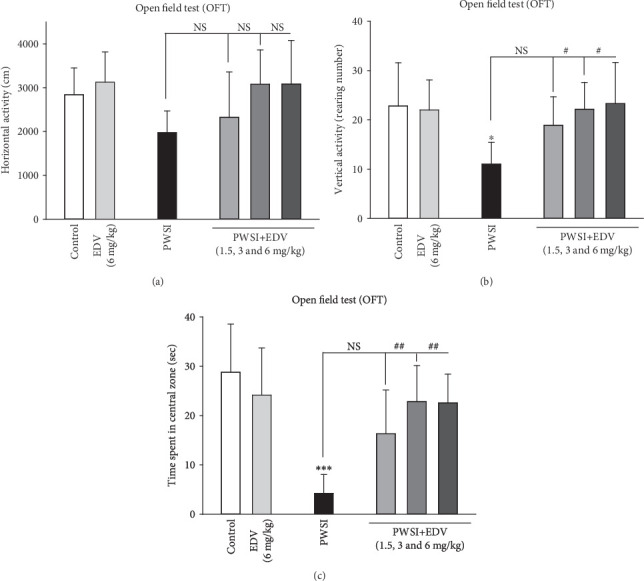
The effect of edaravone (EDV) treatment on (a) horizontal activity, (b) vertical activity, and (c) central zone locomotion. Values are expressed as the mean ± SD and were analyzed using a one-way ANOVA followed by Tukey's post hoc test. A significant difference was accepted (NS = not significant; ⁣^∗^#*P* < 0.05; ⁣^∗∗^##*P* < 0.01; ⁣^∗∗∗^###*P* < 0.001) (*n* = 8–10).

One-way ANOVA analysis showed significant effects of PWSI induction for horizontal activity (*F*(5, 36) = 2.574; *P* = 0.043), vertical activity (*F*(5, 36) = 3.435; *P* = 0.012), and CZL (*F*(5, 36) = 8.439; *P* < 0.001), respectively. As Figures 2(a) and 2(b) depict, the socially isolated rodents showed a significant drop in vertical activity and CZL (*P* < 0.05). However, neither isolation nor treatment groups did not alter locomotor activity in any significant pattern (*P* > 0.05). However, edaravone treatment at 3, 6 mg/kg managed to improve the vertical and CLZ when compared to social isolated group (*P* < 0.05). In any of the three aforementioned factors, the application of 6 mg/kg edaravone did not demonstrate any significant alteration (*P* > 0.05; Figures 2(a)–2(c)). Furthermore, time spent in central zone locomotion, which is an anxiety pattern in rodents, was measured in OFT. Data revealed a significant reduction in time spent in the central zone in PWSI rats (*F*(5, 36) = 8.438; *P* < 0.001; Figure 2(c)). On the other hand, treatment with EDV (1.5–6 mg/kg) showed significant recovery of the anxiogenic effect in PWSI rats (*P* < 0.001). Also, EDV (6 mg/kg) treated in normal rats did not show any alteration in time spent in the central zone (*P* > 0.05; Figure 2(c)).

### 3.2. Edaravone Ameliorated Depressive-Like and Anhedonic Behavior in FST and Splash Test

**Figure 3:**
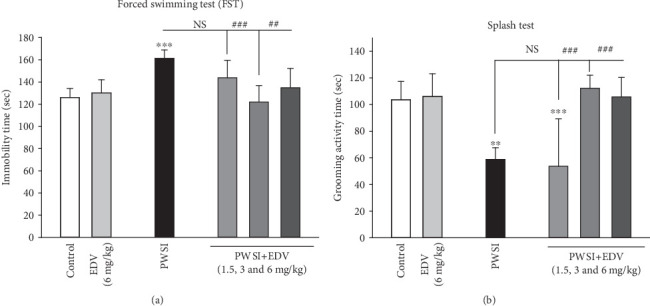
The effect of edaravone (EDV) treatment on (a) force swimming and (b) splash test results. Values are expressed as the mean ± SD and were analyzed using a one-way ANOVA followed by Tukey's post hoc test. A significant difference was accepted (NS = not significant; ⁣^∗^#*P* < 0.05; ⁣^∗∗^##*P* < 0.01; ⁣^∗∗∗^###*P* < 0.001) (*n* = 8–10).

One-way ANOVA analysis showed significant effects of PWSI induction for the FST (*F*(5, 36) = 8.431; *P* < 0.001) and the splash test (*F*(5, 36) = 13.598; *P* < 0.001), respectively. Figures 3(a) and 3(b) reveal that the PWSI group has demonstrated a significant increase in immobility time and major drop in grooming activity (*P* < 0.05). Subsequent administration of edaravone managed to decrease immobility, demonstrating the highest effect at 3 mg/kg compared to the PWSI group (*P* < 0.001). Alterations in grooming activity were also noticed by administration of edaravone, by showing the highest recovering effect at 3 and 6 mg/kg groups compared to the PWSI group (*P* < 0.001). The injection of 6 mg/kg edaravone did not alter immobility time or grooming activity (*P* > 0.05).

### 3.3. Edaravone Alleviated Anxiety Pattern Induced by SI in EPM

**Figure 4:**
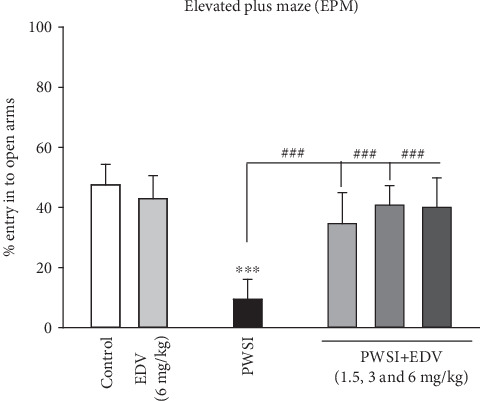
The effect of edaravone (EDV) treatment on elevated plus maze test results. Values are expressed as the mean ± SD and were analyzed using a one-way ANOVA followed by Tukey's post hoc test. Significant difference was accepted (⁣^∗^#*P* < 0.05; ⁣^∗∗^##*P* < 0.01; ⁣^∗∗∗^###*P* < 0.001) (*n* = 8–10).

One-way ANOVA analysis showed significant effects of PWSI induction (*F*(5, 36) = 19.74; *P* < 0.001). Results of Figure 4 indicated that the socially isolated group demonstrated significantly higher tend to stay in close arms compared to control groups (*P* < 0.001). All treatment doses of edaravone managed to significantly recover the anxiogenic effect of SI in a significant pattern when compared to the PWSI group (*P* < 0.001). The EDV 6 mg/kg group did not demonstrate any alteration in the percentage of their entry into open arms (*P* > 0.05).

### 3.4. Edaravone Inhibited the Peroxidation of Lipids and Carboxylation of Proteins

**Figure 5:**
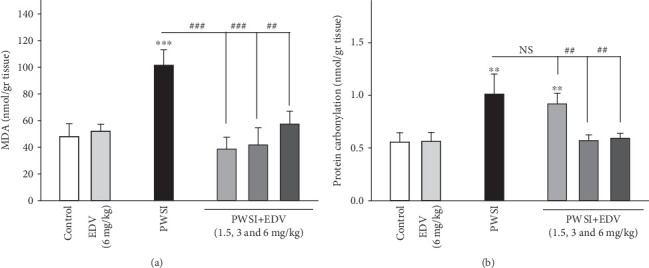
The effect of edaravone (EDV) treatment on (a) MDA level and (b) protein carbonylation amount. Values are expressed as the mean ± SD and were analyzed using a one-way ANOVA followed by Tukey's post hoc test. A significant difference was accepted (NS = not significant; ⁣^∗^#*P* < 0.05; ⁣^∗∗^##*P* < 0.01; ⁣^∗∗∗^###*P* < 0.001) (*n* = 3).

One-way ANOVA analysis showed significant effects of PWSI induction for the MDA (*F*(5, 15) = 19.654; *P* < 0.001) and the PCO (*F*(5, 15) = 12.68; *P* < 0.001), respectively. As displayed in Figures 5(a) and 5(b), both MDA and PCO were significantly increased by the impediment isolation method in rodents (*P* < 0.01). Edaravone managed to modulate the cellular accumulation of PCO and MDA while demonstrating a significant decrease in the overproduction of MDA at all applied doses when compared to the control group (*P* < 0.01), and the same pattern was applied for PCO at treatment doses of 3 and 6 mg/kg (*P* < 0.01). The EDV group did not demonstrate any alteration in MDA and PCO markers (*P* > 0.05).

### 3.5. Edaravone Treatment Led to the Recovery of Total Cellular Antioxidants and GSH Resources

**Figure 6:**
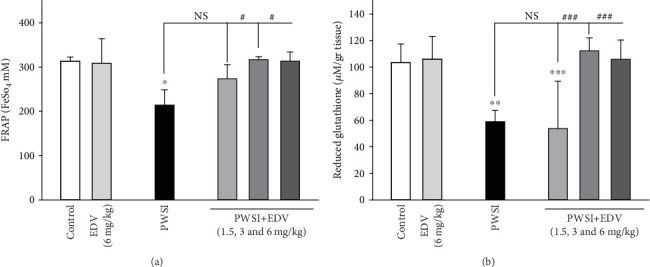
The effect of edaravone (EDV) treatment on (a) total antioxidant level and (b) GSH level. Values are expressed as the mean ± SD and were analyzed using a one-way ANOVA followed by Tukey's post hoc test. A significant difference was accepted (NS = not significant; ⁣^∗^#*P* < 0.05; ⁣^∗∗^##*P* < 0.01; ⁣^∗∗∗^###*P* < 0.001) (*n* = 3).

One-way ANOVA analysis showed significant effects of PWSI induction for the FRAP (*F*(5, 15) = 6.922; *P* < 0.001)and the GSH (*F*(5, 15) = 7.31; *P* < 0.001), respectively. As presented in Figures 6(a) and 6(b), a noticeable drop in FRAP and GSH levels was observed as a result of SI compared to the control group (*P* < 0.05). The PSWI+EDV group demonstrated a significant increase in FRAP and GSH factors in treating doses of 3 and 6 mg/kg compared to the PSWI group (*P* < 0.001 for the GSH and *P* < 0.05 for the TCA, respectively). No significant alterations in FRAP and GSH were noticed in the EDV (6 mg/kg) group compared to the control group (*P* > 0.05).

### 3.6. Edaravone Managed to Regulate the Expression of AMPK, Tlr-4, BDNF, nNOS, and iNOS

One-way ANOVA analysis showed significant effects of PWSI induction for the AMPK (*F*(5, 12) = 71.491; *P* < 0.001), Tlr-4 (*F*(5, 12) = 103.046; *P* < 0.001), BDNF (*F*(5, 12) = 39.107; *P* < 0.001), nNOS (*F*(5, 12) = 26.53; *P* < 0.001), and iNOS (*F*(5, 12) = 21.836; *P* < 0.001), respectively.

**Figure 7:**
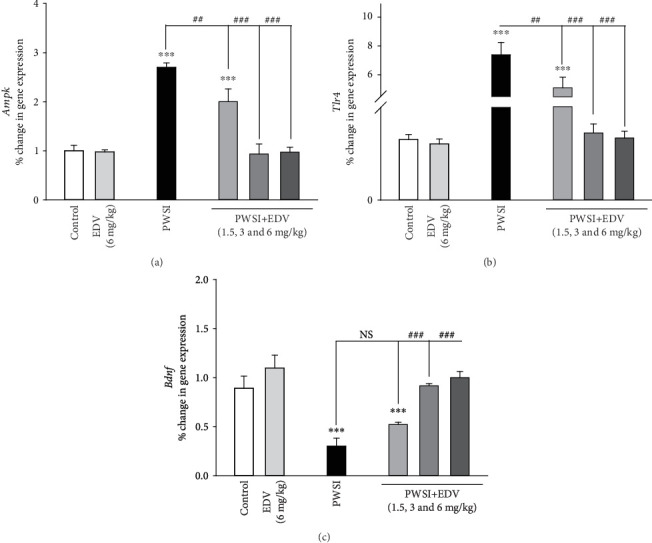
The effect of Edaravone (EDV) treatment on (a) AMPK, (b) Tlr-4 and (c) BDNF expression level. Values are expressed as the mean ± SD and were analyzed using one-way ANOVA followed by Tukey's post hoc test. Significant difference was accepted at (NS = not significant, ⁣^∗^#p <0.05, ⁣^∗∗^##p <0.01, ⁣^∗∗∗^###p <0.001) (n =3).

As the results of Figures 7(a) illustrate, AMPK gene expression is one of the markers demonstrating cellular energy homeostasis. The PWSI significantly increased AMPK expression compared to the control group (*P* < 0.001). Subsequent treatment with edaravone normalized AMPK expression by showing a significant drop in expression in the 3 and 6 mg/kg treatment groups compared to the PWSI group (*P* < 0.001). 6 mg/kg of edaravone application imposed no alteration in the expression of AMPK (*P* > 0.05).

The Toll-like receptor (Tlr-4) gene is mainly contributed in the onset of the immunological response against free radical accumulation. SI has caused a significant increase in expression of Tlr-4 (*P* < 0.001; Figure 7(b)), while treatment at the doses of 3 and 6 mg/kg has significantly ameliorated the overexpression of Tlr-4 in comparison to the PWSI group (*P* < 0.001). 6 mg/kg of edaravone did not change Tlr-4 expression significantly (*P* > 0.05).

BDNF decreases exposure to pathophysiologially damaging substances. In this study, the applied PWSI procedure significantly depressed the expression of BDNF (*P* < 0.001; Figure 7(c)). The groups which were treated with 3 and 6 mg/kg of edaravone recovered the normal level of expression compared with the PWSI group (*P* < 0.001). The injection of edaravone on rats with normal social experience has not altered the BDNF expression compared to the control group (*P* > 0.05).

**Figure 8:**
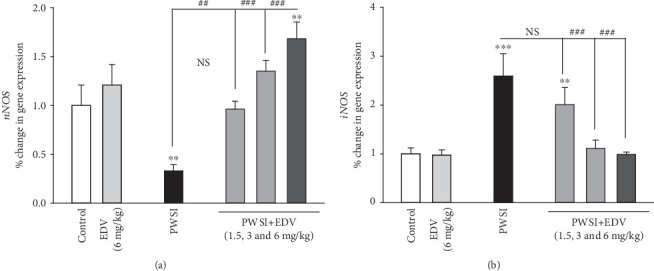
The effect of edaravone (EDV) treatment on (a) nNOS and (b) iNOS expression levels. Values are expressed as the mean ± SD and were analyzed using a one-way ANOVA followed by Tukey's post hoc test. Significant difference was accepted (NS = not significant; ⁣^∗^#*P* < 0.05; ⁣^∗∗^##*P* < 0.01; ⁣^∗∗∗^###*P* < 0.001) (*n* = 3).

nNOS expression can regulate HPA-axis performance [28]. While the PWSI group showed significantly less expression level for nNOS compared to the control group (*P* < 0.01; Figure 8(a)), the PWSI+EDV group has a significantly higher nNOS expression compared to the PWSI group (*P* < 0.01). The PWSI+EDV 6 mg/kg has demonstrated a significantly higher level of nNOS expression compared to the control (*P* < 0.01). 6 mg/kg of edaravone has not significantly altered nNOS expression compared to the control (*P* > 0.05).

The inflammatory mediator escalation could impact the expression of iNOS. In the PWSI group, the level of iNOS expression was significantly higher than the control group (*P* < 0.001; Figure 8(b)). 3 and 6 mg/kg edaravone treatment significantly suppressed the overexpression of iNOS in compare with the PWSI (*P* < 0.001). The EDV (6 mg/kg) group showed no significant change in iNOS expression compared to the control (*P* > 0.05).

### 3.7. Edaravone Can Improve Tissue's Histopathological Properties

**Figure 9:**
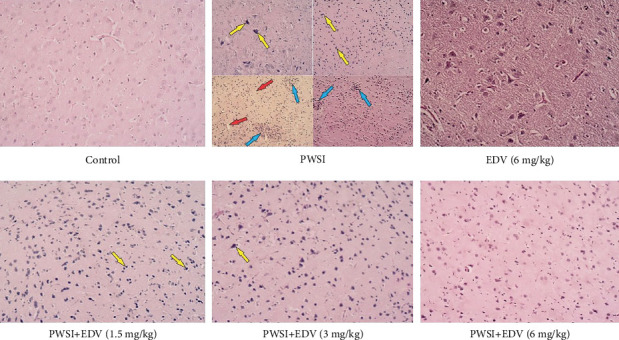
A demonstration of edaravone (EDV) treatment on hippocampal histopathology.

As presented in Figure 9 and the grading on Table 2, following PWSI induction, the basophilic necrosis, vacuolation, and accumulation of inflammatory cells were present. Following treatment with 1/5 and 3 mg/kg of edaravone, mild basophilic necrosis was observed.

## 4. Discussion

By imposing PWSI on Wistar rats, they have shown depressive-like behaviors, as presented in the results of the OFT, FST, EPM and splash tests. The edaravone treatment mainly at doses of 3 and 6 m/kg has ameliorated the behavioral dysfunctions, while it recovered the level of the GSH and FRAP and suppressing the surge of PCO and MDA. This improvement was correlated with the modulation of Tlr-4, AMPK, BDNF, iNOS, and nNOS pathways [18, 29, 30].

In our previous study, applying eight weeks of PWSI environment on rodents has caused significant mitochondrial dysfunction as well as inhibited the maturation of the prefrontal cortex leading to the manifestation of schizophrenic-like disorder in the hippocampus of male and female Wistar rats during adolescence [31]. In this study, shorter period of isolation has caused depressive-like disorder by the means of behavior and biochemical alterations. Neuroinflammation methods of depressive-like behavior induction provided by intracerebral injection of substances like streptozotocin and lipopolysaccharide are based on neuroinflammation and an increase of proinflammatory cytokines which we utilized in our previous study [21]. The utilized method for SI has a long-lasting effect through the adulthood of rats demonstrating highly similar resemblance to disconnecting the attachment insecurity in humans known to be in connection with cholinergic signaling in the hippocampus [32].

In response to social stress, many neurochemical, neuroendocrine, and immune factor responses may occur. Our results demonstrated a significant drop in BDNF expression and an increase in expression of AMPK and Tlr-4 as a response to social stress exposure. AMPK activation is known to be in correlation with the AMP:ATP ratio, which rises when the ATP production system malfunctions. Higher BDNF expression has a direct impact in neurogenesis and cellular plasticity. In addition, there is a direct correlation between overexpression of AMPK and downregulation of BDNF, which could lead to the activation of NMDA channels and a rise in glutamate cellular level [33].

TLRs have an important role in recognizing and initiating the immune response and inflammation. A rise in proinflammatory cytokines can drastically affect Tlr-4 expression. This factor's expression represents direct impact on NF-*κβ*, TLR7/8 pathways, ion channel routes, MAPK, and MyD88 pathways, imposing neuroinflammation and neural damage [34]. This could be a correlation between the stimulating effect of SI on the HPA-axis and the overexpression of Tlr-4 [35].

SI also induced suppressive effect on the level of cellular antioxidants and GSH amount which may be in connection to the overproduction and accumulation of ROS manifesting itself through markers like PCO and MDA which observed through our previous studies [36].

In conclusion, the SI model represents many aspects of depression, including behavioral impairment, neuroinflammation, and immune response similar to markers noticed in human depressive disorder. Making it a considerable choice to investigate edaravone as an intervention.

In our study, application of edaravone mainly at higher treated doses (3 and 6 mg/kg) mitigated anxiety-like disorder by normalizing vertical activity and CLZ in OFT and recovering the EPM activity significantly compared to the PWSI group. In one study, 3 mg/kg edaravone when subjected to a middle cerebral artery occlusion model for ischemia, managed to improve the vertical activity and velocity of rodents' in OFT and the distance ratio in open arms in EPM when compared to the middle cerebral artery occlusion-induced rodent model, demonstrating similar results as saffron [37].

Edaravone can ameliorate depressive-like and anhedonic-like behaviors by the means of improved performance in the FST and splash test. In a premier study that has been approved, the ameliorating effect of edaravone treatment on chronic social defeat stress (CSDS) by means of modulating Nrf2 expression, astrocyte dysfunction, and preventing microglial activation prevented CSDS-induced behavioral dysfunction [29].

On a cellular scale, administration of edaravone in the PWSI model has recovered shortage in GSH and FRAP resources which by observation of PCO and MDA results, these effects could cause by free radical scavenging properties of edaravone in a dose-dependent pattern [38]. The aforementioned effect would lead to improvement of mitochondrial performance and restoration of cellular ATP production [39].

The AMPK pathway acts as a marker in demonstrating cellular homeostasis status, yet it is observed that accumulation of reactive oxygen species would cause overexpression of the AMPK gene [40]. Deregulation of insulin-dependent glucose transport activity in the brain was also noticed to be in correlation with AMPK activity in the SI model [41]. The overexpression of AMPK was arrested by the administration of edaravone at 3 and 6 mg/kg. MDA and AMPK modulations could confirm the edaravone effect in normalizing neural cell homeostasis and mitochondrial activity in the PWSI model.

3 and 6 mg/kg of Edaravone treated rats demonstrated a significantly decreased Tlr4 and an increased BDNF expression. While Tlr-4 overexpression could lead to an increase in immune system response, activation of BDNF gene would decrease neruoinflammation and facilitate the process of neural tissue recovery, preventing cell loss [42].

Nitric oxide production in the brain is mainly impacted by mood disorders, while SI caused downregulation in expression of nNOS and upregulation of iNOS, causing the accumulation of degraded cells in the hippocampus and a high presence of inflammatory cells and microglial activation. The consequent treatment with edaravone has significantly modulated the expression of iNOS and nNOS, while downregulating Tlr-4 and upregulating BDNF, leading to improvement in neural elasticity and function. The regulatory effect of edaravone on iNOS and TNF expression during the surge of proinflammatory has been observed in past studies [43]. However, the modulatory effect of edaravone treatment on inflammatory, neurotrophic, antioxidant, and energy production markers while imposing no toxic effect at any treatment dose in a socially isolated rodent model is the novel aspect of this study.

## 5. Conclusion

The imposing of SI environment on rodents has caused depression, lack of motivation, and anxiety-like disorders which accompanied by the surge in oxidative stress markers, namely, MDA and PCO. Edaravone treatment ameliorates behavioral impairment and suppressed oxidative stress markers level. In addition, edaravone demonstrated significant modulating effect on expression of Tlr-4, AMPK, BDNF, iNOS, and nNOS, which contribute to neuroinflammation, energy production, and neural tissue recovery.

## Figures and Tables

**Table 1:** Primer sequences used for real-time PCR assay.

Name	Sequence (5′ ⟶3′)	Genbank
*Ampk*	ACCTGAGAACGTCCTGCTTG GGCCTGCGTACAATCTTCCT	NM_001013367.3
*Tlr4*	GATCTGAGCTTCAACCCCTTG TGCCATGCCTTGTCTTCAAT	NM_021297.3
*Bdnf*	ATCCACTGAGCAAAGCCGAA CCTGGTGGAACATTGTGGCT	NM_012513.4
*nNOS*	CTGACCTGTTGCTTAGGGATA TCATCTGCTCATTGCCATTCG	NM_039089061.1
*iNOS*	AGCCACGGATATTTAGAGTG CAGAGAAAGAGCACATAGAC	NM_039085203.1
*β Actin*	CTAGGCACCAGGGTGTGATG GCACAGGGTGCTCCTCAG	NM_007393.5

**Table 2:** Grading of histopathological changes in the brain in the control and experimental rats. Scoring was done as follows: NO (0); Mild (1); Moderate (2); and Severe (3). Microglial nodule (blue arrows), basophilic necrotic neuron (yellow arrows), and vacuolization (red arrows) were observed.

Groups	Microglial nodule	Basophilic necrotic neuron	Vacuolization
Control	0	0	0
PWSI	2	2	2
EDV (6 mg/kg)	0	0	0
PWSI+EDV (1.5 mg/kg)	0	1	0
PWSI+EDV (3 mg/kg)	0	1	0
PWSI+EDV (6 mg/kg)	0	0	0

## Data Availability

The data used to support the findings of this study are available from the corresponding author upon request.

## Abbreviations

EDV: Edaravone
*AMPK*: AMP-activated protein kinase
ATP: Adenosine triphosphate
*BDNF*: Brain-derived neurotrophic factor
DTNB: 5,5′-Dithiobis (2-nitrobenzoic acid)
EPM: Elevated plus maze
FRAP: Ferric ion reducing antioxidant power
FST: Forced swimming test
GSH: Glutathione
LPO: Lipid peroxidation
PCO: Protein carbonylation
MDA: Malondialdehyde
*nNOS*: Neuronal nitric oxide synthase
*iNOS*: Inducible nitric oxide synthase
OFT: Open field test
PWSI: Post weaning social isolation
HPA: Pituitary and adrenal axis
Tlr4: Toll-like receptor 4
ROS: Reactive oxygen species.
